# Concomitant pulmonary and hepatic toxicity secondary to nitrofurantoin: a case report

**DOI:** 10.1186/1752-1947-1-59

**Published:** 2007-08-01

**Authors:** Adrian F Peall, Aidan Hodges

**Affiliations:** 1 Respiratory Department, Hawke's Bay Hospital, Hastings, New Zealand

## Abstract

**Background:**

Concomitant pulmonary and hepatic toxicity secondary to nitrofurantoin is a rare but serious complication of the use of Nitrofurantoin.

**Case presentation:**

A 72 year old woman taking Nitrofurantoin for recurrent urinary sepsis presenting with breathlessness abdominal discomfort and abnormal liver function tests is described. Drug toxicity secondary to Nitrofurantoin was diagnosed. Cessation of the drug and a course of steroids markedly improved her condition.

**Discussion:**

We review the drug reactions associated with Nitrofurantoin and suggest an alternative treatment strategy for recurrent urinary sepsis.

**Conclusion:**

Adverse drug reactions are an important cause of concomitant lung and liver toxicity and the mainstay of treatment is drug withdrawal.

## Background

Nitrofurantoin is well recognized as a cause of adverse drug reactions although the combination of lung and liver toxicity is rare [1]. A case of concomitant lung and liver toxicity in a patient taking nitrofurantoin and our approach to management is described. We discuss the presentation of nitrofurantoin induced drug reactions and suggest an alternative treatment strategy for patients taking this drug for recurrent urinary tract infections.

## Case presentation

A 72 year old retired woman presented to her General Practitioner with breathlessness, upper abdominal discomfort and nausea. She was treated initially for a lower respiratory tract infection with a five day course of amoxicillin and prednisone. Blood analysis at this time revealed abnormal liver function tests (LFTs) [see additional file 1]. Her breathlessness continued to worsen after this initial course of therapy and she was admitted for further investigation and treatment.

A review of her history in the months prior to this acute deterioration revealed increasing breathlessness, not relieved by her usual medication, forcing her to give up her favourite walking routes. Past medical history included stable asthma managed by her general practitioner, recurrent urinary tract infection and osteoporosis. She had no drug allergies and her prescribed medications were: zopiclone 7.5 mg od; medroxyprogesterone acetate 5 mg od; conjugated oestrogens 0.3 mg od; amitriptyline 10 mg od; nitrofurantoin 100 mg tds; fluticasone 250 μg inhaled od; naproxen 550 mg bd; hyoscine butylbromide 10 mg as required; paracetamol 1 gm as required up; and salbutamol 200 μg inhaled as required. The dose of nitrofurantoin had been increased 5 months prior to admission from 100 mg od, she had been taking it for 6 months prior to this dose increase. She had no risk factors for development of liver pathology, did not smoke and rarely drank alcohol.

On examination there were no signs of liver disease. Her abdomen was soft with mild right upper quadrant tenderness. Respiratory examination was unremarkable with no audible wheeze or crepitations, oxygen saturations were 97% at rest. The remaining examination did not reveal any further signs and of note there was nothing to suggest an underlying autoimmune disease.

A full blood count revealed a haemoglobin of 159 g/L (normal 115–155) with no other abnormalities. Repeat LFTs had deteriorated [see additional file 1] the remaining biochemistry was normal. Chest radiograph demonstrated bilateral interstitial and alveolar shadowing in the middle and lower zones. Upper abdominal ultrasound scan demonstrated an enlarged hypoechoic liver.

A high index of suspicion of an adverse drug reaction at this point lead to cessation of nitrofurantoin. In addition she was commenced on a course of oral corticosteroids; Prednisone 40 mg od as an adjunct to cessation of the nitrofurantoin.

A full screen for liver disease delivered the following abnormal results; IgG was elevated at 15.0 g/L (normal 7.0–14.0). Anti-nuclear antibody was positive (diffuse, chromosome positive pattern) at a titre of 1/80. Anti-smooth muscle antibody was positive at a titre of 1/40. Epstein Barr virus and Cytomegalovirus serology were both consistent with past infection.

**Figure 1:**
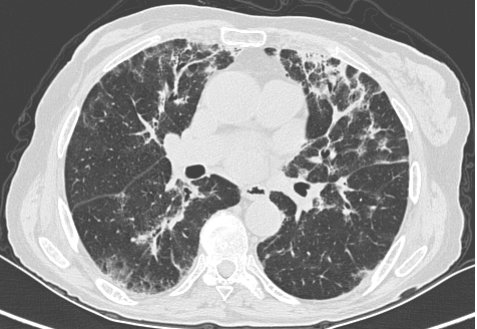
High resolution computer tomogram of the chest showing peripheral alveolar shadowing and fibrotic changes.

Pulmonary function tests demonstrated impaired diffusion capacity of 0.70 mmol/min/kPa/L (49% predicted) FEV1 2.64, FVC 3.30, FEV1/FVC 79.92%. High resolution computer tomogram of the chest revealed peripheral alveolar shadowing and fibrotic changes [see Fig. 1]. A liver biopsy was performed which showed moderate lobular hepatitis characterised by zone 3 necrosis, which was considered to be consistent with a drug induced hepatitis.

After withdrawal of nitrofurantoin and commencement of oral corticosteroids this woman made a good symptomatic recovery. At follow up LFTs had normalized [see additional file 1] and diffusion capacity had increased to 0.83 mmol/min/kPa/L (58% predicted) her FEV1 was 2.97 and FVC 3.71 (FEV1/FVC 80.13%). Her chest radiograph was reported as normal at follow up. Her prednisone was stopped after an 8 week course

Two years following her initial presentation and cessation of nitrofurantoin she has had no recurrence of her symptoms.

## Discussion

Nitrofurantoin is used for the prophylaxis and treatment of uncomplicated urinary tract infection. Adverse drug reactions (ADR) to nitrofurantoin include pulmonary reactions, hepatic toxicity, blood dyscrasias and peripheral neuropathy [1]. Concomitant pulmonary and hepatic toxicity secondary to nitrofurantoin is rare with few reported cases [2-5].

We reviewed the possibility that her other medications may have contributed to her combined pulmonary and hepatic pathology. Of her prescribed medications, in addition to nitrofurantoin, only medroxyprogesterone had been reported as causing pulmonary fibrosis and this only in combination with radiotherapy [6]. Drugs causing combined lung and liver pathology are few and include busulfan, chlorambucil, amiodarone, methotrexate as well as nitrofurantoin [5]. With this information we felt that nitrofurantoin was responsible. Additionally, the findings of the liver biopsy were compatible with a drug induced hepatitis adding weight to the diagnosis.

The vast majority of pulmonary reactions to nitrofurantoin (90%) are acute and characterised by fever, cough, dyspnoea, and peripheral eosinophilia [1]. Radiological findings include pulmonary infiltrates which resolve rapidly upon drug withdrawal [1]. Nitrofurantoin also causes a range of chronic pulmonary disease, often presenting with insidious onset of increasing dyspnoea, dry cough and radiological evidence of fibrosis, as in this case [6,7]. Nitrofurantoin is also recognised to produce a picture of hepatocellular toxicity consistent with chronic active hepatitis with elevated ANA, Anti Smooth Muscle antibodies and elevated IgG [2]. Liver biopsy results in this patient are similar to those seen in other reported cases of liver and lung toxicity secondary to nitrofurantoin [4,5].

Initial treatment consists of drug withdrawal. In addition, we elected to use oral corticosteroids, which previous cases have also tried, despite no definite evidence to support their use in addition to withdrawal of nitrofurantoin [5]. Interestingly this woman's symptoms did not improve in response to the short course of prednisone prescribed by her GP whilst still on nitrofurantoin. Ongoing use of the drug would be a factor in refractoriness to steroid treatment [8]. The underlying mechanism behind nitrofurantoin toxicity remains uncertain; an immunological response is suggested by the presence of autoantibodies. Direct cytotoxic mechanisms; for example by increased oxidative stress, have also been suggested [9]. Following drug withdrawal both acute and chronic reactions to nitrofurantoin tend to resolve. Deaths have been reported due to both chronic lung and liver damage although these are rare [1]. Encouragingly even apparently widespread fibrotic changes in the lungs have been seen to resolve after nitrofurantoin withdrawal [10].

The use of nitrofurantoin for UTI prophylaxis in this woman is clearly no longer appropriate. She vehemently agreed with this and self initiated regular intake of dried cranberries. There is some evidence to support her approach [11]. In post-menopausal women topical intravaginal estriol cream has been shown to be an effective treatment for recurrent UTIs as lower post-menopausal oestrogen levels cause vaginal flora changes predisposing to infection [12].

An alternative to her previous prophylactic regime is self-initiated intermittent treatment of UTIs with short course antibiotics [12]. In light of her history this was our recommendation to the patient; she found this an acceptable management plan, as she was keen to minimize her intake of further antibiotics.

## Conclusion

Adverse drug reactions should be considered in patients presenting with concomitant lung and liver toxicity with the mainstay of treatment being drug withdrawal.

## Competing interests

The author(s) declare that they have no competing interests.

## Authors' contributions

AP and AH both contributed equally to the preparation, reading and approval of the manuscript.

## Supplementary Material

Additional file 1 Serial liver function tests, before, at and after admission to hospital. This file shows a graphical representation of liver function tests throughout this patients' management. Click here for file
